# Serum Levels of Eosinophil-Derived Neurotoxin, Platelet-Activating Factor and Vascular Endothelial Growth Factor in Adult Patients with Atopic Dermatitis—A Pilot Study

**DOI:** 10.3390/biomedicines10123109

**Published:** 2022-12-01

**Authors:** Krzysztof Gomułka, Ewa Wójcik, Jacek Cezary Szepietowski

**Affiliations:** 1 Clinical Department of Internal Medicine, Pneumology and Allergology, Wroclaw Medical University, 50-369 Wroclaw, Poland; 2 Clinical Department of Dermatology, Venerology and Allergology, Wroclaw Medical University, 50-368 Wroclaw, Poland

**Keywords:** atopic dermatitis, eosinophil-derived neurotoxin, platelet-activating factor, vascular endothelial growth factor, pruritus

## Abstract

Atopic dermatitis (AD) is a chronic, highly pruritic, relapsing–remitting inflammatory skin disease. The etiology of AD has not been fully explained yet and complex interactions of various small molecules are still being taken into account. The aim of this research was to investigate the serum eosinophil-derived neurotoxin (EDN), platelet activating factor (PAF) and vascular endothelial growth factor (VEGF) concentrations in relation to the disease severity and pruritus intensity in adult patients with AD. This pilot study was performed on 30 participants (15 patients with AD and 15 healthy controls). Blood samples were taken to examine the serum levels of EDN, PAF and VEGF using the enzyme-linked immunosorbent assay (ELISA) test. The severity of disease was assessed by the Scoring Atopic Dermatitis (SCORAD) index. The intensity of pruritus, as a subjective symptom, was determined by the Visual Analogue Scale (VAS). Obtained results revealed that the EDN (*p* = 0.016) and VEGF (*p* = 0.032), but not PAF (*p* = 0.841) concentrations were significantly higher in patients with AD compared with those of the control group. There was positive correlation between the EDN level and the SCORAD index in patients with AD (r = −0.9, *p* = 0.037) which was not found for the PAF and VEGF levels. Circulating EDN, PAF and VEGF levels were not significantly correlated with the severity of pruritus. Our results suggest that the END and VEGF serum levels are significantly increased in patients with AD compared to control group. Moreover, EDN might be useful to reflect the severity of symptoms.

## 1. Introduction

Atopic dermatitis (AD) is a chronic inflammatory skin disease characterized by remitting eczematous lesions and intense itch sensation. The number of patients with AD in children and in the adult population is still considerably high, amounting to about 2–17.6% in various international surveys [1,2,3,4]. The etiology of AD is complex and has not been fully explained yet. However, it might be associated with genetic and environmental factors and has a link with some cytokines taking part in autoimmune inflammation [5,6,7,8]. Dysfunction of the skin barrier related to epidermal defect and Th2-mediated immune response accompanied by the release of multiple molecules from the immune system cells are hallmark elements in pathogenesis of AD in the acute and chronic phases [9,10,11]. In the past, several studies were performed to prove that cytokines orchestrate atopic skin inflammation and investigate the possible association between such cytokines and AD severity. It has been suggested that IL-10, IL-6, IFN-γ and IL-4 levels were significantly decreased in AD group compared with controls; additionally, serum IgE, TNF and YKL-40 levels correlate with AD severity [12,13,14,15]. In our study, we focused on eosinophil-derived neurotoxin, platelet-activating factor and vascular endothelial growth factor—the main objectives were to contribute the serum level of these three molecules and assess the possible correlation with the severity of disease and itch intensity in adult patients with AD in comparison to healthy controls.

Eosinophil-derived neurotoxin (EDN) is an enzyme of the ribonuclease (RNAse) type synthesized by eosinophils and analogous in function to the eosinophil cationic protein. EDN is deposited in the eosinophil granules and its biological activity is focused on neurotoxic effect, cytotoxic impact on bacteria and viruses and chemotaxis induction of certain cells (i.e., monocytes and neutrophiles) [16]. Elevated level of EDN enhances synthesis of matrix metalloproteinase-9, which in turn can reduce eosinophilic chronic inflammation and the remodeling process on the surrounding tissues. What is more, available reports [17,18] have shown that EDN is important in the pathophysiology of asthma, where is associated with the airway hyperresponsiveness and severity of the disease.

Platelet-activating factor (PAF) is a phospholipid continuously produced in a very low concentration by platelets, neutrophils, monocytes, macrophages and endothelial cells. Its synthesis is increased during an inflammatory condition, an allergic response, shock, platelet aggregation and activation of thrombotic cascades or dilation of blood vessels. PAF-acetyl hydrolase (PAF-AH) degrades the PAF and PAF-like phospholipids, controlling their actions. Rupatadine, as a representative of a second-generation antihistamine agonist, affects histamine and PAF pathways, which further inhibits mast cell activation. This dual braking is approved for the treatment of allergic rhinitis and chronic urticaria [19,20].

Vascular endothelial growth factor (VEGF) in terms of chemical structure is a heparin-binding homodimer glycoprotein that plays a pivotal role during the angiogenesis process. Epithelial cells, macrophages, platelets, and neutrophils are mainly VEGF-secreting cells. The “VEGF family” consists of: VEGF-A (simply called VEGF), VEGF-B, VEGF-C, VEGF-D, VEGF-E, VEGF-F and placental growth factor (PlGF). Deregulation in VEGF metabolism may implicate in various diseases such as asthma, chronic obstructive pulmonary disease, age-related macular degeneration, diabetic retinopathy, ischemic heart disease, rheumatoid diseases, cancers and metastasis [21,22,23].

Despite the growing understanding of the pathomechanism of AD, a specific parameter that could easily be measured has not been found so far. To the best of our knowledge, there are not many recent reports concerning the issue of EDN, PAF and VEGF in adult patients with AD showing the correlation with some clinical parameters of the disease. That is why we have decided to explore that issue in skin atopy—this is the first study further examining only these three molecules, observed regardless of the presence of other atopic diseases, in the Polish adult population. The significance of this report is to extend our knowledge on EDN, PAF and VEGF in AD, attempt to indicate among them a probable biomarker for AD diagnosing and monitoring and propose as a possible new therapeutic target of the disease.

## 2. Materials and Methods

### 2.1. Examined Groups

This pilot study included a total number of 30 participants: 23 female and 7 male patients, aged from 20 to 49 years (average age: 30.91 ± 8.65 years). The study was approved by the local ethical committee (protocol code KB—224/2020) and all patients signed informed consent to participate in the study. Among them, 15 patients (12 female and 3 male patients, aged from 20 to 49 years (average age: 30 ± 11.31 years)) were diagnosed with atopic dermatitis according to criteria defined by Hanifin and Rajka [24]. Patients with AD were in a stable period of the disease; they only used topical skin care preparations and emollients without taking systemic treatment (the washout period for systemic treatment with long-acting antihistamines or systemic corticosteroids was no less than 14 days before the recruitment). The control group of healthy volunteers consisted of 15 subjects: 11 female and 4 male patients, aged from 21 to 40 years (average age: 31.67 ± 6.77 years). They did not suffer from any chronic skin diseases and did not take permanently treatments for any diseases. The population data of all participants are shown in Table 1.

The exclusion criteria of all participants included: lack of consent, age under 18 and over 70 years, pregnant and breastfeeding female patients, presence of acute skin changes and taking drugs from the group of long-acting antihistamines or systemic corticosteroids up to 14 days prior to the study.

### 2.2. Blood Collection and Biochemical Analysis

A quantity of 5 mL of venous blood samples were taken from all patients and healthy controls. Then, the serum was separated after centrifugation and kept at temperature −70 °C for further research. Next, the mean serum levels of EDN, PAF and VEGF were measured using the human enzyme-linked immunosorbent assay (ELISA) kits, according to the manufacturers’ instructions (Wuhan EIAAB Science Co., Ltd., Wuhan, China).

### 2.3. Disease’s Severity

The Scoring Atopic Dermatitis (SCORAD) index was used for the assessment of disease severity in patients with atopic dermatitis. Based on the obtained number of points, patients can score with mild (<25 points), moderate (25–50 points) or severe (>50 points) AD [25].

The Visual Analogue Scale (VAS) was applied to evaluate the intensity of pruritus from the previous week. Participants were asked to assess intensity of itch, form 0 points (no itching) to 10 points (worst imaginable itch). The results were ranked as follows: 0–2.9 points—mild pruritus; 3–6.9 points—moderate pruritus; 7–8.9 points—severe pruritus and 9–10 points—very severe pruritus [26].

### 2.4. Statistical Analysis

Statistical analysis was performed using the Statistica 13.3 software (StatSoft, Kraków, Poland). The Bartlett test was used to examine the homogeneity of variance. Verification of the hypothesis of equality of mean parameters in independent groups was carried out by the nonparametric Mann–Whitney U test. The χ^2^ test was used to compare nominal and ordinal data. The Spearman correlation coefficient was performed for analysis of selected parameter pairs. A *p* value of less than 0.05 was accepted as statistically significant.

## 3. Results

The obtained results revealed significantly higher mean serum levels of EDN and VEGF in AD patients than in healthy controls. PAF level also was found higher in AD patients, but this difference was not statistically significant.

**Figure 1:**
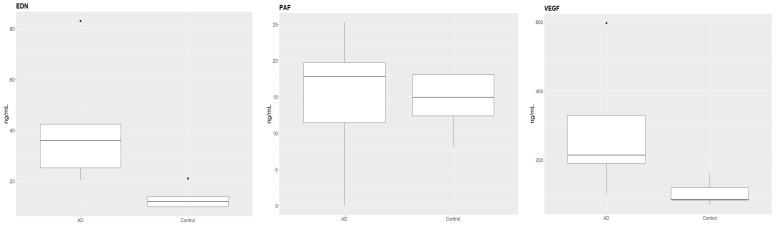
The mean serum concentrations of the examined molecules in patients with atopic dermatitis (AD) and healthy control.

The differences in serum concentrations of examined molecules in both groups of participants are shown on Figure 1 and in Table 2.

In the study group of patients with AD, 3 participants had severe AD, and 12 participants had a moderate degree of disease severity according to the SCORAD index. Furthermore, we found relationships between the mean EDN serum concentration and the severity of AD—there was a significant positive correlation between EDN level and the SCORAD index. On the contrary, no correlation was found between PAF, or VEGF concentration and the intensity of AD measured by SCORAD index. 

All patients with AD reported skin pruritus of average–moderate intensity assessed using VAS scale. Nevertheless, the mean serum EDN, PAF and VEGF concentrations did not correlate with the intensity of itch sensations. 

The data about disease severity in our patients with AD are shown in Table 3.

## 4. Discussion

Our study supports the immune and inflammatory component in AD mediated largely by selected chemokines whose potential role in the pathogenesis of the disease is indicated by an increased serum level. We demonstrated that serum levels of all three investigated molecules in patients suffering from AD were higher than in group of healthy controls—it may indicate their important role in the complex inflammatory process observed in patients with AD. In accordance with studies from available literature, many cellular mediators, i.e., bioactive lipids, leukotrienes, prostaglandins, cytokines or proteases are critical regulators of signaling cascades, degranulation and chemotaxis in the course of not only atopic dermatitis, but also of asthma and rhinitis, food allergies, anaphylaxis, and urticaria [27,28,29]. In other places, a possible role of cells like eosinophils and basophils in the pathology of skin disorders has been described, indicating that mediators are synthesized and released in inflammation after cell activation during AD. Some molecules, among others EDN, PAF and VEGF, have been demonstrated in lesioned atopic dermatitis skin, where they might reflect a migratory response with basophils and eosinophils “priming”, and consequently have an irritating impact on surrounding tissues [30,31]. In our study, marked differences in EDN, PAF and VEGF concentrations between AD patients and healthy participants also seem to confirm the cytokine imbalance as a part of the pathogenesis of AD. In several studies conducted (e.g., by Jenerowicz et al. [32] and Taniuchi et al. [33]), the mean level of eosinophil proteins including EDN in serum and urine of AD patients has been found to be significantly increased compared with the healthy controls. Like our findings, these results suggest the usefulness of EDN as a part of diagnostic approach to AD patients. Moreover, it has been previously shown that EDN might be related to the disease severity. In examined AD patients, Kim et al. [34] revealed the serum levels of EDN were significantly higher in the severe recalcitrant AD group and severe AD group compared with the mild to moderate AD group. Additionally, EDN had a significant positive correlation with the SCORAD index. Furthermore, it has been reported by Goto et al. [35] that the urinary EDN concentration in patients with AD was correlated with visual analogue scale (VAS scores) for itching, skin condition or overall skin symptoms. According to the above data and our present results, we conclude that EDN may strongly reflect disease severity and could be a biomarker for evaluating AD disease activity.

According to data from the literature, the histological features of the subacute or chronic stages of AD are connected to angiogenesis and linked to pro-angiogenic factors, including VEGF, which was found increased in the stratum corneum of patients with AD compared to non-lesional skin. Additionally, the mean serum level of VEGF was found to be significantly higher in AD patients than in the control group, but there was not a significant correlation with disease severity [12,36], which stays in agreement with our present results.

However, in contrast to our findings, Samochocki et al. [12] and Lee et al. [37] described that the VEGF concentrations correlated with the severity of AD as expressed by the SCORAD index. Nevertheless, considering that VEGF showed a significant decrease after systemic treatment with methotrexate or azathioprine in patients with AD [15], this molecule could be regarded as a potentially important mediator in the pathogenesis of AD, a practicable biomarker correlating with severity and therapeutic response in patients with AD.

A few studies with in vitro and in vivo analysis concluded that PAF, as a member from the group of lipid mediators, might have an impact in the immunopathogenesis of AD [38]. Interestingly, the authors have suggested that colonization by *Staphylococcus aureus* is associated with exacerbation of AD induced by eosinophil activation via the PAF receptor [39]. Additionally, the findings of Jenks et al. [40] indicate that PAF induced opening of endothelial gaps and extravasation of neutrophils and eosinophils in the normal-appearing skin of patients with AD. Moreover, a study with a topically applied PAF antagonist was performed in patients with AD but the therapeutic efficacy did not demonstrate a superior effect in comparison to the placebo [41]. Taken together, previous reports in the literature about PAF and our findings showing that PAF level was detected in higher concentrations in systemic circulation in patients with AD than in control group, it can be asserted that this molecule is important in skin inflammation and AD pathogenesis. A correlation that was not statistically significant was obtained between high PAF concentration and the intensity of AD measured with the SCORAD index. This supports the notion that definitions of the cytokine profiles and mechanisms underlying such an elevation in AD patients are still not clear.

Results from our study give information that may be useful for better understanding of the potential role of EDN, PAF and VEGF in patients with AD. However, it has not been definitively clear whether the examined molecules are associated with chronic inflammation in the course of AD or if are involved in specific biochemical reactions during atopy. Moreover, in the obtained results, there was no correlation between these molecules’ levels and the pruritus intensity in AD patients.

Pruritus represents one of the most bothersome symptoms (that is uncomfortable sensation underlying the desire to scratch), but in most cases, it causes sleep disturbances and reduces overall quality of life. Its pathogenesis is diverse and involves a complex histaminergic and non-histaminergic pathway, where the crosstalk between the immune system, cutaneous and neuronal cells has an impact on numerous dermatological and systemic diseases. Among the known pruritogens that signal through shared Janus kinase pathways, the most important roles are played by Th2 cytokines (IL-4, IL-13, IL-31), thymic stromal lymphopoietin, periostin, substance P, neurokinin 1R, neurotrophins nerve growth factor, autotaxin, histamine H4 receptor and oncostatin M [42,43,44]. In the inflamed lesional skin of AD patients compared to healthy controls, cells of the inflammatory, including eosinophils, basophils and lymphocytes, infiltrate and have been described to release various pruritogens and through their interaction with neuronal cells, which is correlated with itch severity. For individuals with AD, increased blood eosinophils and basophils may serve as a biomarker of T helper cell type 2 polarization through the secretion of various cytokines, including histamine, IL-31 or periostin, and are likely to contribute to itch [31,45,46]. Considering our research, it should be noted that VEGF is known to be increased in AD lesions which correlates with local vascular abnormalities, trans-epidermal water loss and skin–water content. What is more, VEGF over-expression with angiogenesis and increasing vascular permeability in AD patients, may induce itch indirectly by recruiting many immune cells (i.e., eosinophils and basophils) that target pruritogenic substances to lesional sites [47,48]. Additionally, the PAF produced by a variety of cells is involved in increasing vascular permeability causing activation and migration of eosinophils, neutrophils, and monocytes, leading to increased and prolonged skin inflammation with itch. In AD patients, PAF also activates mast cells by peripheral nerve activation, then with degranulation and secretion of a wide variety of itch-related mediators, including histamine. For this reason, addition of anti-PAF action to the H1-receptor agonist reduces itching more effectively [31,49]. Furthermore, it has been suggested that eosinophil-derived proteins in serum may reflect the inflammatory activities of eosinophils in allergic diseases, and in study by Goto et al. [35], urinary EDN concentrations in patients with AD did not correlate with involvement of eosinophilic inflammation in the formation of atopic dry skin with itching.

The lack of positive correlation between molecule serum levels and itch intensity may suggest it plays a supportive, rather than a key, role in the pathogenesis of pruritus in the course of AD; however, further studies on a larger group of patients are required to confirm these observations.

Some limitations of our pilot study should be pointed out: small sample size, no dividing AD patients into subtypes (intrinsic vs. extrinsic), no relations between other biochemical markers (i.e., IgE level, CCL17 and CCL18) and no other scores for AD severity (i.e., EASI) have been investigated. Nevertheless, it should be emphasized that our work is the first to consider these three small molecules simultaneously and the first to be carried out in the adult Polish population.

## 5. Conclusions

In summary, our results support the concept of a systemic and an inflammatory component in AD. The presented pilot study is one of the reports to simultaneously assess an increased serum level of EDN, PAF and VEGF in adult patients with AD. Moreover, EDN and VEGF levels were significantly higher in AD patients compared to the control group, but only the EDN level was relevant to disease severity by the SCORAD index. Therefore, the findings described above may be useful for establishing these small molecules being involved in the complex etiology of the disease and as a potential factor playing a role in the immunopathological progression of AD. In addition, the presented study puts emphasis on the possible supportive role of EDN, PAF and VEGF in the pathogenesis of pruritus during AD. Nevertheless, more research is needed on a larger sample of patients to clarify whether EDN, PAF, and VEGF may be prognostic tools prior to AD exacerbations, biomarkers of this disease or new effective targeted therapies.

## Figures and Tables

**Table 1:** Demographic data of the examined groups.

Parameters	Atopic Dermatitis Group	Control Group
Participants, *n*	15	15
Age (years), mean ± SD	30 ± 11.31	31.67 ± 6.77
Sex female, *n* (%)	12 (80%)	11 (73.33%)
BMI (kg/m^2^), mean ± SD	27.8 ± 6.3	26.7 ± 6.8
Smoker—current or former, *n* (%)	8 (53.33%)	6 (40%)
Other concomitant atopic disease: asthma, *n* (%)	2 (13.33%)	1 (6.67%)
Other concomitant atopic disease: rhinitis, *n* (%)	3 (20%)	2 (13.33%)

BMI—Body Mass Index; SD—standard deviation.

**Table 2:** Laboratory findings in examined participants.

Parameters	Atopic Dermatitis Group (*n* = 15)		Control Group (*n* = 15)		*p* Value
	$\overset{´}{x}\pm \;\mathrm{SD}\;(\mathrm{Range})$	Minimal–Maximal Value	$\overset{´}{x}\pm \;\mathrm{SD}\;(\mathrm{Range})$	Minimal–Maximal Value	
EDN (ng/mL)	35.9 ± 24.89	20.24–83.05	12.0 ± 4.61	9.77–20.99	0.016
PAF (ng/mL)	17.8 ± 9.66	0.0–25.27	14.9 ± 4.19	8.13–18.05	0.841
VEGF (ng/mL)	213.7 ± 192.59	98.67–597.47	84.6 ± 36.83	69.36–160.53	0.032

$\overset{´}{x}$—mean; SD—Standard Deviation; EDN—Eosinophil-Derived Neurotoxin; PAF—Platelet-Activating Factor; VEGF—Vascular Endothelial Growth Factor.

**Table 3:** Clinical characteristic of the examined patients with AD.

Parameters	Atopic Dermatitis Group (*n* = 15)		Correlation		
	$\overset{´}{x}\pm \;\mathrm{SD}\;(\mathrm{Range})$	Minimal–Maximal Value	EDN	PAF	VEGF
SCORAD index (points)	40.6 ± 17.20	26–70	r = −0.9 *p* = 0.037	r = 0.2 *p* = 0.747	r = 0.6 *p* = 0.285
Pruritus (VAS) (points)	6.0 ± 2.12	4–9	r = 0.1 *p* = 0.784	r = −0.02 *p* = 0.959	r = 0.29 *p* = 0.411

$\overset{´}{x}$—Mean; SD—Standard Deviation; SCORAD—The Scoring Atopic Dermatitis Scale; VAS—The Visual Analogue Scale; EDN—Eosinophil-Derived Neurotoxin; PAF—Platelet-Activating Factor; VEGF—Vascular Endothelial Growth Factor.

## Data Availability

The data that support the findings of this study are available from the corresponding author upon request.
